# Contributory Factors to Successful Tuberculosis Treatment in Southwest Nigeria: A Cross-Sectional Study

**DOI:** 10.3390/tropicalmed7080194

**Published:** 2022-08-19

**Authors:** Olanrewaju Oladimeji, Kelechi Elizabeth Oladimeji, Mirabel Nanjoh, Lucas Banda, Olukayode Ademola Adeleke, Teke Apalata, Jabu Mbokazi, Francis Leonard Mpotte Hyera

**Affiliations:** 1 Department of Public Health, Faculty of Health Sciences, Walter Sisulu University, Mthatha 5117, Eastern Cape, South Africa; 2 Department of Laboratory Medicine and Pathology, Faculty of Health Sciences, Walter Sisulu University, Mthatha 5117, Eastern Cape, South Africa; 3 Medical Education Unit, Faculty of Health Sciences, Walter Sisulu University, Mthatha 5117, Eastern Cape, South Africa; 4 Department of Family Medicine, Walter Sisulu University, Mthatha 5117, Eastern Cape, South Africa; 5 Office of the Dean, Faculty of Health Sciences, Walter Sisulu University, Mthatha 5117, Eastern Cape, South Africa

**Keywords:** tuberculosis, multidrug-resistant (MDR) strains, successful treatment outcome

## Abstract

Tuberculosis (TB) is one of the oldest human diseases, and preventing treatment failure is critical. This is because TB cases pose a risk to the immediate and remote communities due to the potential for spread, particularly for multidrug-resistant (MDR) strains that have been associated with higher morbidity and mortality rates. Hence, this study looked at the factors that influence TB treatment outcomes in Southwest Nigeria. We conducted a cross-sectional study with 712 TB patients from 25 directly observed treatment short course (DOTS) centers, out of which 566 (79.49%) were new treatment cases, and 102 (14.33%) were retreatment cases. The outcome variable was computed into successful treatment where ‘Yes’ was assigned to TB treatment completed and cured, and ‘No’ was assigned to all the remaining outcomes following the standard TB definition. Independent variables included in the analysis were the patient’s socio-demographic characteristics (such as age, sex, distance from the facility, marital status, family type, education, and computed socioeconomic status from modified DHS household assets), clinical and facility parameters (such as the HIV status, facility of access to healthcare, healthcare workers attitudes, services offered at the facility, appearance of the facility, number of people seeking care and waiting time at the facility). Bivariate analysis showed that HIV status (OR: 3.53, 95% CI: 1.83–6.82; *p* = 0.001), healthcare worker attitude (OR: 2.13, 95% CI: 1.21–3.74; *p* = 0.01), services offered at the facility (OR: 0.67, 95% CI: 0.49–0.92; *p* = 0.01), appearance of facility (OR: 0.67, 95% CI: 0.46–0.98; *p* = 0.04), and number of people seeking care (OR: 2.47, 95% CI: 1.72–3.55; *p* = 0.001) were associated with higher odds of successful treatment outcome with statistical significance. After multivariate analysis, reactive HIV status (aOR: 3.37, 95% CI: 1.67–6.80; *p* = 0.001), positive attitude of healthcare workers (aOR: 2.58, 95% CI: 1.36–4.89; *p* = 0.04), excellent services offered at the healthcare facility (aOR: 0.53, 95% CI: 0.36–0.78; *p* = 0.001) and few people seeking care (aOR: 2.10, 95% CI: 1.21–3.84; *p* = 0.001) became independent significant determinants of successful treatment outcome. The study concluded that reactive HIV status, positive attitude of healthcare workers, few people seeking healthcare, and excellent service provided were all factors that contributed to successful treatment outcomes.

## 1. Introduction

Tuberculosis remains a major public health concern, particularly in underdeveloped countries with weaker health systems [1]. As a result, more than 95% of global TB cases and deaths occur in this region, further eroding individuals’ livelihoods [2]. Tuberculosis is also a threat in Nigeria, contributing to the disease’s high mortality rate. Since the beginning of the AIDS epidemic, the prevalence of tuberculosis in adults has increased [3]. According to the Global TB Report, 10 countries account for 77% of the global TB case detection gap, and Nigeria is in the second position after India [4].

Tuberculosis (TB) is a highly contagious disease mainly caused by Mycobacterium tuberculosis (MTB) and typically affects the lungs (pulmonary tuberculosis) and other parts of the body (extrapulmonary tuberculosis) [5]. General symptoms included fatigue, weight loss, fever, loss of appetite, chills, and night sweats [6]. The disease is spread by airborne droplet nuclei in the air when infected people expel the bacteria through coughing or sneezing [7]. Symptoms that go unnoticed in the early stages of a TB disease are more likely to cause a severe and more difficult to treat case. They increase both morbidity and mortality and pose a risk to infection control [8,9]. 

A greater risk of infection has been observed in individuals with constant and prolonged close contact with individuals already infected with TB [10,11,12]. It is known that the very young and elderly are at increased risk of TB transmission and progression, although most cases of TB occur in adults as risk increases with age [13,14]. In addition, people with lower socioeconomic status, people living in less ventilated areas, and marginalized populations, especially those in prison, are at higher risk of contracting TB, mainly due to crowded living conditions and coinfection with HIV [15,16].

In most African countries, up to 80% of TB patients are HIV seropositive, suggesting that TB is the most important opportunistic infection in HIV-infected individuals worldwide [16,17]. This makes HIV a major determining factor for TB treatment outcome and vice versa. HIV seropositive individuals are more likely to have active tuberculosis and progress to AIDS [18,19]. Kwan and Ernst called the convergence of TB and HIV coinfection a syndemic [19]. According to the World Health Organization, a total of 1.5 million people died from tuberculosis worldwide in 2018 (including 251,000 people living with HIV). The 30 countries with a high TB burden accounted for 87% of new TB cases, eight of which account for two-thirds of the total, with India leading the tally, followed by China, Indonesia, the Philippines, Pakistan, Nigeria, Bangladesh, and South Africa [20]. According to the Global TB Report 2019, Africa has the highest TB burden, with Nigeria experiencing the increasing burden [21]. Compared to the 2018 report, TB incidence in Nigeria increased from 418,000 cases in 2017 to 429,000 cases in 2018, with deaths from 155,000 to 157,000, while treatment coverage for TB stagnated at 24% [21].

While the number of laboratory-confirmed cases of drug-resistant tuberculosis has decreased, with incidence falling from 2300 cases in 2017 to 2275 cases in 2018, the projected cases have increased from 5400 in 2017 to 21 000 in 2018 [22]. Notably, the majority (about 75%) of the cases occur among the most economically productive age group, resulting in substantial economic loss [23]. Furthermore, the increasing rates of multidrug-resistant (MDR)-TB and TB/HIV coinfection further strain public health resources and complicate TB control efforts [24]. However, there has been progress in the percentage of HIV-positive people receiving tuberculosis preventive medication, which has risen from 39% in 2017 to 62% in 2019 [22].

In 1993, the World Health Organization (WHO) declared TB a global public health emergency. In 1995, the directly observed treatment short course (DOTS) regimen was adopted as the key strategy in resolving this global problem [25]. By 2005, 4.9 million cases of TB patients had been treated using DOTS in 187 countries [26], including Nigeria [27]. The strategy has been shown to be effective in achieving a highly successful treatment outcome and has become an essential indicator in evaluating the effectiveness of the tuberculosis control program [28]. One of the major privileges of the DOTS program in Nigeria is that treatment is provided free of charge to TB patients [29,30].

Early detection of tuberculosis and prompt initiation of treatment have been found to contribute to successful treatment. Late TB diagnosis could lead to increased infectivity, disease burden, and death [31,32]. Although there is efficacious chemotherapy for TB, therapy requires greater than 90% adherence to facilitate treatment success and reduce the emergence of multidrug TB (MDR-TB) [32]. Further, treatment interruption is a significant obstacle in TB control, and the reasons for this temporary termination are complex. They include patients’ characteristics and income, the socio-cultural context, TB’s chronic nature, and patients’ relationship with health care workers [33,34]. Consequently, this study aimed to assess the drivers of successful treatment outcomes for TB in Oyo State, Southwest Nigeria.

## 2. Materials and Methods

### 2.1. Study Setting

Ibadan is the capital city of Oyo State, in Nigeria, with over 3 million people [35]. There are eleven local government areas in Ibadan, consisting of five urban local governments in the city and six semi-urban local governments in the peri-urban communities. The most dominant tribe in Ibadan is the Yorubas [36].

### 2.2. Study Design and Sampling

A cross-sectional study was conducted among 712 TB patients in twenty-five (25) DOT centers using a semi-structured interviewer-administered questionnaire. This study was conducted from June to October 2016. Eligible participants recruited included all TB patients treated at all selected DOTs centers.

### 2.3. Inclusion Criteria

All TB patients who consented to participate in the study.

Patients age 18 years and above.

### 2.4. Exclusion Criteria

These are TB patients who do not provide their consent to participate in the study.

### 2.5. Clinical Management of Susceptible TB Patients

The National Tuberculosis and Leprosy Control Program (NTBLCP) diagnostic algorithm encourages a suspected TB case to undergo multiple bacteriological investigations (i.e., acid fast bacilli (AFB)-swab microscopy or/and Xpert MTB/RIF) for early diagnosis [37]. This process usually begins with screening for cough of more than two weeks duration, with or without weight loss or/and night sweats or/and fever, and is then evaluated for TB [37]. Two sputum samples are submitted for AFB smear microscopy using Ziehl-Neelsen staining methods. Extrapulmonary TB (EPTB) is diagnosed based on clinical or laboratory findings, and the clinician makes an assessment. All patients diagnosed with tuberculosis are also counseled and tested for HIV [37].

Drug-sensitive TB treatment consists of two phases: the two-month intensive treatment phase and the four-month continuation phase for new patients. In accordance with WHO guidelines, the current anti-tuberculosis regimen begins with a two-month intensive phase treatment with four fixed-dose combinations (FDC), namely rifampicin (R), isoniazid (H), pyrazinamide (Z), and ethambutol (E), and four-month follow-up phase with two FDC (isoniazid and ethambutol) (i.e., 2(RHZE)/4(EH)) [37,38]. Rifabutin (Rfb) is used in place of rifampicin for HIV/AID co-infected TB patients on second-line antiretroviral therapy [37]. Directly observed treatment (DOT) is observed throughout the period, either through direct supervision by DOT staff or through the involvement of treatment supporters [37]. The operational definitions of tuberculosis treatment outcomes are provided in Table 1.

### 2.6. Variables of Interest

The outcome variable was computed into successful treatment where ‘Yes’ was assigned to TB treatment completed and cured, and ‘No’ was assigned to all the remaining outcomes following the standard TB definition [37,38,39].

Independent variables included in the analysis were the patient’s socio-demographic characteristics (such as age, sex, distance from the facility, marital status, family type, education, and computed socioeconomic status from modified DHS household assets [40]) and clinical and facility parameters (such as the HIV status, facility of access to healthcare, healthcare workers attitudes, services offered at the facility, appearance of the facility, number of people seeking care and waiting time at the facility).

### 2.7. Data Analysis

Descriptive statistics were employed to describe the respondent’s trajectory (the number of times patients have received TB treatment) by socio-demographic and health and healthcare facility factors. The association between the outcome and independent variables was assessed by employing a multivariate binomial backward stepwise logistic regression model. Odds ratios (OR) with 95% confidence intervals (CIs) and *p*-values ≤ 0.05 were reported for all statistically significant results. All statistical analyses were performed using Stata software version 12 (Stata Corp, College Station, TX, USA).

## 3. Results

### 3.1. Socio-Demographic Characteristics of Participants

Overall, a total of 712 participants were recruited for the study. The majority were 31–40 years, 30.10% (87), females, 61.74% (439), secondary level of education, 41.99% (299), and of middle social economic status, 58.85% (419). Almost two-thirds of the participants, 73.46% (523), live within 10 km distance from the health facility they take treatment. Almost 70% (495) of the respondents were married or cohabiting, with the majority in a monogamous home 64.99% (453), as shown in Table 2.

### 3.2. Clinical Characteristics of the Study Participants

The overwhelming majority, 89.60% (629), of the participants use the government health facility as their primary place of access to healthcare, are new treatment cases, 79.49% (566), and have non-reactive status for HIV, 86.22% (582). Two-fifths, 41.89% (284), had a successful treatment outcome, while more than half, 58.11% (394), had unsuccessful treatment outcomes. Among the participants, those that take their drugs daily were in the majority 73.31% (522), and among the major reasons why they attended a primary healthcare center for their previous treatment contact were trusted, 39.47% (281), belief, 29.92% (213), and proximity, 29.63% (211). Most of the participants stayed six months and more at their first treatment site, 56.74% (404), experienced less than 30 min waiting time, 82.33% (559), received counseling as a service, 74.02% (527), and were of the opinion that the healthcare worker’s attitude was positive, 89.33% (611), as shown in Table 3.

### 3.3. Socio-Demographic and Clinical Characteristics Associated with Successful Treatment Outcome

The bivariate analysis showed that HIV status (OR: 3.53, 95% CI: 1.83–6.82; *p* = 0.001), healthcare worker attitude (OR: 2.13, 95% CI: 1.21–3.74; *p* = 0.01), and number of people seeking care (OR: 2.47, 95% CI: 1.72–3.55; *p* = 0.001) were associated with successful treatment outcome with statistical significance. Other variables that had an association with successful treatment outcomes with statistical significance were services offered at the healthcare facility (OR: 0.67, 95% CI: 0.49–0.92; *p* = 0.01) and appearance at the healthcare facility (OR: 0.67, 95% CI: 0.46–0.98; *p* = 0.04), as shown in Table 4.

Multiple logistic regression analysis was performed to control confounding variables after which significant determinants of treatment outcome were reactive HIV status (aOR: 3.37, 95% CI: 1.67–6.80; *p* = 0.001), positive healthcare worker attitude (aOR: 2.58, 95% CI: 1.36–4.89; *p* = 0.04), excellent services offered at the healthcare facility (aOR: 0.53, 95% CI: 0.36–0.78; *p* = 0.001), and few people seeking care (aOR: 2.10, 95% CI: 1.21–3.84; *p* = 0.001), as shown in Table 5.

## 4. Discussion

The findings of this research shed light on what makes a TB treatment successful. Successful treatment outcomes were linked to a positive healthcare worker attitude, HIV status, the presence of a low number of patients seeking care, excellent service, and an attractive healthcare facility.

Although age has no significant relationship with successful treatment outcomes in this study, assessment of the age distribution shows that the TB burden was most prevalent in young adults and middle-aged people. Many researchers have reported comparable results [41,42,43]. Evidence has shown that the burden of TB across age groups differs by geographical setting. In a study conducted in China, TB cases were estimated to occur in people aged 45 and up [44], while in the countries within Africa, such as findings of another study conducted in Kenya [45], the majority (40.9%) of the TB/HIV cases were in the 25–34-year age bracket, with children under the age of 15 accounting for 4.9% of the cases. These findings on differing TB burdens across age groups are also consistent with the World Health Organization’s global, African, and Nigerian estimates [2]. Plausible causal factors for this trend have been attributed to a high prevalence of youthful exuberance, instability, and drug use among this group, making it difficult for them to remain in care after being diagnosed with TB (ref). Consequently, the young age group should be an important target for public health measures, and it is essential to possess extensive knowledge about them [46].

Another finding in this study was that females sought treatment 1.5 times more frequently than males. Women in Sub-Saharan Africa account for 63% of all new HIV infections in 2020, making them the most at risk of developing active TB and more likely to develop drug resistance [47]. Doctor in Malawi [48] and Kongolo in South Africa [49] observed that women’s status as belonging to the vulnerable group with poverty, malnourishment, overcrowding, and poor housing makes them more susceptible to TB. This finding is comparable to that of Dogar et al. [48] Pakistan study, which found that the proportion of female TB cases reported in the western provinces is roughly double that of the eastern provinces and Pakistan. However, this contradicts the findings of other studies in which males predominate [41,42,43,49], including a Nigerian study conducted by Effiong et al. [50]. Males had a significantly higher prevalence of tuberculosis than females, according to Nyamogoba and Mbuthia [45].

Barely than 40% of the participants in this study had a successful treatment outcome, defined as those who completed their treatment and those who were declared cured. This is significantly lower than the WHO’s global treatment success estimates of 75% and 86% for Nigeria in 2019 [2], as well as the results of some studies conducted in India [42], Ethiopia [51], Denmark [43], Brazil [52], and Nigeria [41]. It highlights the fact that millions of patients with TB, particularly in Africa, do not have unrestricted access to effective anti-TB drugs. The high treatment success observed in a similar study in the same region in Nigeria by Sunday et al. [41] may be due to the full supervision of the DOTS strategy in the treatment centers. In addition, the high treatment success observed in a study conducted in Cotonou, Benin Republic, was due to a functioning National Tuberculosis Programme (NTP), adequate drug supply to avoid shortages, strict supervision of drug taking, and a suitable treatment plan for follow-up [53].

The HIV status of the participants revealed that 86% were negative, which is higher than the national estimate of 76% and supports the reported national incidence rate of decrease [54]. The majority of participants chose the primary healthcare center because of its proximity to their place of residence and their trust and belief in the healthcare workers in the facilities. This highlights the positive impact of patient-centered care, respect, and non-stigmatization of patients with TB and ensures patient trust in healthcare providers to improve treatment success rates. Furthermore, the majority acknowledged excellent appearance, positive attitude of healthcare workers, and less than 30 min of waiting time at the center where treatment was received. However, just over half of those polled agreed that their services are excellent. This could be one of the primary reasons for the participants’ treatment success rate of 40%.

The aforementioned predictors of successful treatment outcomes, which included reactive HIV status, a positive attitude among healthcare workers, and a low number of people seeking care at the healthcare center, are similar to findings from the study by Tola et al. [55] and Zeenebe et al. [56]. Patients with TB who had reactive HIV screening are nearly four times more likely to have successful treatment outcomes. This aligns with the findings of Oladimeji et al. [57], Onyeonoro et al. [58], and Ibrahim et al. [59]. Excellent service rendered and the appearance of the healthcare center were two other factors with a weak association with successful treatment outcomes.

After controlling for confounders, the predictive determinants of successful treatment outcomes were reactive HIV status, positive healthcare worker attitude, excellent service at the facility, and a low number of people seeking healthcare. This means that people with HIV coinfection have more than three times the chances of successfully completing treatment, regardless of healthcare workers’ attitudes or the number of people seeking care at the facility. Furthermore, those who experience a positive attitude of healthcare workers and the presence of few people seeking healthcare have twice the likelihood of recording successful treatment, with each determinant independent of the other. It is well understood that having both TB and HIV implies a poor prognosis. This could be explained by their adherence to their treatment regimen and successful treatment.

## 5. Limitations

The study did not examine some clinical characteristics of the patients, such as weight and height, that could be used to assess one of the key indicators of clinical improvement. In addition, the study is limited by not exploring socio-cultural variables that can impact the outcome of treatment.

## 6. Conclusions, Program, and Policy Recommendations

The study concluded that reactive HIV status, positive attitude of healthcare workers, few people seeking healthcare, and excellent service provided were all factors that contributed to successful treatment outcomes. With a poor treatment success rate, there is a need to understand these factors. It is therefore recommended that improved policy on patient-centered care, including upgrading healthcare facilities and continuous training of healthcare workers, should be provided.

## Figures and Tables

**Table 1:** Operational Definitions of Treatment Outcomes.

Treatment Outcome	Operational Definition
Cured	A pulmonary TB patient with bacteriologically confirmed (smear or culture positive) tuberculosis at the beginning of treatment and who was smear or culture-negative in the last month of treatment and on at least one previous occasion [37,38].
Treatment completed	A TB patient who completed treatment but without any evidence of cure or failure (there is no record to show that sputum smear or culture results in the last month of treatment and on at least one previous occasion are negative either because they were not done or results were not available) [37,38].
Treatment failure	A TB patient whose sputum smear or culture is positive at month 5 or later during treatment [37,38].
Died	A TB patient who dies for any reason before or during the course of treatment [37,38].
Lost to follow-up	A TB patient who did not start treatment or whose treatment was interrupted for 2 consecutive months or more [37,38].
Not evaluated	A TB patient for whom no treatment outcome is assigned. This includes cases “transferred out” to another treatment unit and where the treatment outcome is unknown to the reporting unit [37,38].
Treatment success	The sum of bacteriologically diagnosed TB cases cured and those who completed their treatment without a bacteriologically confirmed register [37,38].

**Table 2:** Socio-demographic characteristics of the study participants (n = 712).

Variables	Frequency	Percentage (%)
Age (years) (n = 681)		
<20	56	8.22
21–30	189	27.75
31–40	205	30.10
41–50	115	16.89
51–60	69	10.13
60+	47	6.90
Sex (n = 711)		
Male	272	38.26
Female	439	61.74
Distance from facility (n = 712)		
<5 km	261	36.66
5–10 km	262	36.80
>10 km	189	26.54
Marital status (n = 712)		
Never married	185	25.98
Married/Cohabiting	495	69.52
Divorced/Widowed	32	4.49
Family type (n = 697)		
Monogamous	453	64.99
Polygamous	244	35.01
Education (n = 712)		
Primary and below	232	32.58
Secondary	299	41.99
Tertiary education	181	25.42
Socioeconomic status (n = 712)		
Low SES	231	32.44
Middle SES	419	58.85
Upper SES	62	8.71

**Table 3:** Clinical characteristics of the study participants (n = 712).

Variables	Frequency	Percentage (%)
Place of access to healthcare (n = 702)		
Private health facility	73	10.40
Government health facility	629	89.60
Treatment status		
Retreatment	102	14.33
Relapse	44	6.18
New treatment	566	79.49
Treatment outcome (n = 678)		
Successful	284	41.89
Unsuccessful	394	58.11
HIV status (n = 675)		
Reactive	50	7.41
Non-reactive	582	86.22
Don’t know	43	6.37
How regular patient-administered their drugs (n = 712)		
Never	77	10.81
Daily	522	73.31
Twice a week	22	3.09
Thrice a week	12	1.69
Weekly	63	8.85
Monthly	16	2.25
Facility for the first treatment (n = 712)		
Never	86	12.08
Religious/Traditional center	21	2.95
Private hospital	109	15.31
Primary healthcare center	405	56.88
General/Teaching hospital	91	12.78
Reason for choosing the first treatment facility (n = 712)		
Proximity	211	29.63
Trust	281	39.47
Cost	7	0.98
Believe	213	29.92
Length of treatment at the first treatment facility (n = 712)		
2 months or less	211	29.63
3–5 months	97	13.62
6 months or more	404	56.74
Services received at the first treatment facility (n = 712)		
Counseling	527	74.02
Diagnosis	38	5.34
Treatment	53	7.44
Referral	1	0.14
Support	2	0.28
Combination of counseling, diagnosis, and treatment	24	3.37
Combination of counseling, diagnosis, treatment, and referral	67	9.41
Healthcare worker attitude (n = 684)		
Positive healthcare worker attitude	611	89.33
Not positive	73	10.67
Services offered at the facility (n = 692)		
Excellent	410	59.25
Not excellent	282	40.75
The appearance of the facility (n = 691)		
Excellent	542	78.44
Not excellent	149	21.56
Number of people seeking care (n = 687)		
Few	169	24.60
Many	518	75.40
Waiting time at the facility (n = 679)		
Less than 30 min	559	82.33
More than 30 min	120	17.67

**Table 4:** Factors associated with the number of TB treatments received among the respondents stratified by treatment outcome using bivariate logistic regression.

Variables	Successful Treatment Outcome		cOR (95% CI)	*p*-Value
	Yes n = 284	No n = 394		
	Freq. (%) [95% CI]	Freq. (%) [95% CI]		
Age group				
≤20 ^R^	15 (5.45) [3.08–8.84]	35 (9.38) [7.53–13.41]	-	
21–30	81 (29.45) [24.13–35.23]	103 (27.61) [22.54–31.10]	2.23 (0.96–5.18)	0.061
31–40	83 (30.18) 24.81–35.98]	113 (30.29) [25.79–34.68]	1.22 (0.63–2.34)	0.557
41–50	48 (17.45) [13.16–22.47]	63 (16.89) [13.21–20.42]	1.30 (0.68–2.49)	0.426
51–60	26 (9.45) [6.27–13.55]	36 (9.65) [7.96–13.96]	1.26 (0.63–2.52)	0.521
60+	22 (8.0) [5.08–11.86]	23 (6.17) [4.21–8.93]	1.32 (0.61–2.87)	0.476
Sex				
Male	106 (37.32) [31.68–43.23]	153 (38.93) [34.37–43.58]	-	
Female	178 (62.68) [56.77–68.32]	240 (61.07) [56.42–65.63]	0.93 (0.68–1.28)	0.671
Distance from facility				
<5 km ^R^	95 (33.45) [27.99–39.27]	154 (39.09) [34.29–43.48]	-	
5–10 km	114 (40.14) [34.39–46.10]	133 (33.76) [30.23–39.20]	1.14 (0.77–1.68)	0.521
>10 km	75 (26.41) [21.38–31.94]	107 (27.16) [22.67–31.02]	0.82 (0.56–1.20)	0.308
Marital status				
Never married ^R^	63 (22.18) [17.49–27.47]	113 (28.68) [24.43–32.96]	-	
Married/Cohabiting	210 (73.94) [68.43–78.95]	261 (66.24) [61.99–70.89]	0.99 (0.44–2.19)	0.973
Divorced/Widowed	11 (3.87) [1.95–6.82]	20 (5.08) [3.23–7.38]	0.68 (0.32–1.46)	0.325
Family type				
Monogamous ^R^	185 (66.55) [60.67–72.07]	248 (64.42) [59.26–68.41]	-	
Polygamous	93 (33.45) [27.93–39.33]	137 (35.58) [31.59–40.74]	1.09 (0.79–1.52)	0.569
Education				
Primary ^R^	77 (27.11) [22.03–32.68]	140 (35.53) [31.80–40.87]	-	
Secondary	134 (47.18) [41.26–53.17]	155 (39.34) [34.06–43.24]	1.34 (0.89–2.02)	0.162
Tertiary education	73 (25.70) [20.72–31.20]	99 (25.13) [21.35–29.56]	0.85 (0.58–1.25)	0.413
Socioeconomic status				
Low SES ^R^	76 (26.76) [21.70–32.31]	143 (36.29) [31.80–40.87]	-	
Middle SES	182 (64.08) [58.20–69.67]	216 (54.82) [50.64–60.01]	1.39 (0.78–2.49)	0.257
Upper SES	26 (9.15) [6.07–13.13]	35 (8.88) [6.14–11.43]	0.88 (0.51–1.52)	0.650
HIV status				
Reactive	35 (12.82) [9.09–17.38]	13 (4.0) [2.55–6.78]	-	
Non-reactive	238 (87.18) [82.62–90.91]	312 (96.0) [93.22–97.45]	3.53 (1.83–6.82)	0.001 *
Place of access to healthcare				
Private health facility	26 (9.15) [6.07–13.13]	47 (12.24) [8.56–14.63]	-	
Government health facility	258 (90.85) [86.87–93.93]	337 (87.76) [85.37–91.44]	0.72 (0.44–1.20)	0.208
Healthcare worker attitude				
Positive healthcare worker attitude	259 (93.50) [89.92–96.10]	325 (87.13) [82.82–89.47]	-	
Not positive	18 (6.50) [3.90–10.08]	48 (12.87) [10.53–17.18]	2.13 (1.21–3.74)	0.01 *
Services offered at the facility				
Excellent	151 (54.12) [48.08–60.08]	242 (63.85) [57.95–67.24]	-	
Not excellent	128 (45.88) [39.92–51.92]	137 (36.15) [32.76–42.05]	0.67 (0.49–0.92)	0.01 *
Appearance of facility				
Excellent	208 (74.82) [69.29–79.81]	309 (81.53) [76.80–84.37]	-	
Not excellent	70 (25.18) [20.19–30.71]	70 (18.47) [15.63–23.20]	0.67 (0.46–0.98)	0.04 *
Number of people seeking care				
Few	96 (34.53) [28.95–40.44]	66 (17.60) [14.44–21.85]	-	
Many	182 (65.47) [59.56–71.05]	309 (82.93) [78.15–85.56]	2.47 (1.72–3.55)	0.001 *
Waiting time at the facility				
Less than 30 min	231 (84.93) [80.11–88.96]	297 (79.62) [76.47–84.14]	-	
More than 30 min	41 (15.07) [11.04–19.89]	76 (20.38) [15.86–23.53]	1.44 (0.95–2.19)	0.09

* Statistically significant (*p* < 0.05); R = reference values; cOR = crude odds ratio.

**Table 5:** Factors associated with the number of TB treatments received among the respondents stratified by treatment outcome using multivariate logistic regression.

Variables	Successful Treatment Outcome		aOR (95% CI)	*p*-Value
	Yes n = 284	No n = 394		
	Freq. (%) [95% CI]	Freq. (%) [95% CI]		
HIV status				
Reactive	35 (12.82) [9.09–17.38]	13 (4.0) [2.55–6.78]	-	0.001 *
Non-reactive	238 (87.18) [82.62–90.91]	312 (96.0) [93.22–97.45]	3.37 (1.67–6.80)	
Healthcare worker attitude				
Positive healthcare worker attitude	259 (93.50) [89.92–96.10]	325 (87.13) [82.82–89.47]	-	0.04 *
Not positive	18 (6.50) [3.90–10.08]	48 (12.87) [10.53–17.18]	2.58 (1.36–4.89)	
Services offered at the facility				
Excellent	151 (54.12) [48.08–60.08]	242 (63.85) [57.95–67.24]	-	0.001 *
Not excellent	128 (45.88) [39.92–51.92]	137 (36.15) [32.76–42.05]	0.53 (0.36–0.78)	
Appearance of facility				
Excellent	208 (74.82) [69.29–79.81]	309 (81.53) [76.80–84.37]	-	0.608
Not excellent	70 (25.18) [20.19–30.71]	70 (18.47) [15.63–23.20]	0.88 (0.55–1.42)	
Number of people seeking care				
Few	96 (34.53) [28.95–40.44]	66 (17.60) [14.44–21.85]	-	0.001 *
Many	182 (65.47) [59.56–71.05]	309 (82.93) [78.15–85.56]	2.10 (1.21–3.84)	

* Statistically significant (*p* < 0.05); aOR = adjusted odds ratio.

## Data Availability

The data set produced by the current study is available from the corresponding author upon request.
